# Modeling of the Performance Loss due to Catalyst Deactivation in Fixed- and Fluidized-Bed Reactors

**DOI:** 10.1021/acs.iecr.5c02248

**Published:** 2025-08-26

**Authors:** M. Andrea Pappagallo, Tilman J. Schildhauer, Oliver Kröcher, Emanuele Moioli

**Affiliations:** † Center for Energy and Environmental Science, 28498 Paul Scherrer Institut, Villigen 5232, Switzerland; ‡ Institute of Chemical Sciences and Engineering, École Polytechnique Fédérale de Lausanne, Lausanne 1015, Switzerland; § Dipartimento di Chimica, Materiali e Ingegneria Chimica “Giulio Natta”, 18981 Politecnico di Milano, Milano 20133, Italy

## Abstract

A method to assess the impact of deactivation phenomena on the global performance of a catalytic reactor was developed. The methodology is applied here to the case of CO methanation, where the catalyst is subject to deactivation by coking. This method can be extended to other reactions and deactivation mechanisms. The method is based on the integration of a single differential equation to describe the activity of the catalyst and on the evaluation of the profiles in the reactor through consecutive steady states at progressively lower activity values. The model was applied successfully to both fixed- and fluidized-bed methanation, with small differences between the two cases. This model showed promising results in a case study, with a correct description of the decrease in the level of CO conversion due to coking. It also allowed us to observe the higher resistance to deactivation of fluidized-bed reactors compared to fixed-bed ones at similar conditions. The time needed to reach 25% conversion in fluidized-bed reactors was calculated to be 5 to 50 times higher compared to that in fixed-bed reactors. The model allows optimizing the reactor with respect to deactivation, acting on the reactor geometry, size, and operating conditions to achieve the best long-term performance.

## Graphical abstract

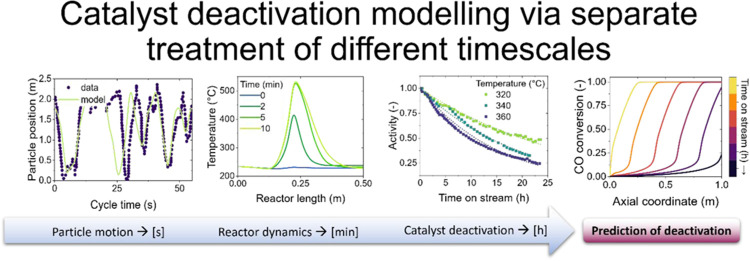

## Introduction

The increasing concerns about the scarcity of resources and about climate change are a major driver toward the research in the field of circular economy and in the reutilization of waste. This involves the development of a variety of new technologies aiming at the reutilization of waste resources. Among them, a special focus goes toward the abatement of greenhouse gas emissions or their collection from the atmosphere and reutilization. The utilization of CO_2_ for the production of chemicals, fuels, and materials is among the most attractive options to reach a carbon-neutral future economy. CO_2_ can be captured either chemically, for example through direct air capture,^1^ or by biomass during its growth.^2^ In the latter case, biomass can then be turned into a mixture of carbon-containing gases (commonly referred to as syngas) through a variety of thermal processes.^3^ If fuels are produced from syngas,^4,5^ their use for energy production will produce the same amount of CO_2_ that was initially captured, leading to an overall carbon-neutral cycle.^6,7^

Most of the CO_2_ and syngas conversion processes are catalytic, and they are affected by the presence of impurities in the feed streams, which can degrade the catalyst activity. Carbon-containing species themselves can represent a danger for the catalyst, as they tend to decompose into quasi-elemental carbon, which deposits on the active surface of the catalyst leading to a loss of activity.^8,9^ The carbon deposits have a variety of molecular structures, depending on the local conditions (atomic H/C ratio, temperature, etc.), on the catalyst composition, on the nature of the reactants and other carbonaceous species involved, and on several other factors. Deactivation by coking has been shown to be reversible since the carbon layer can react in the presence of hydrogen or water and desorb from the catalytic surface at temperatures over 400 °C.^10,11^ Steam addition is employed industrially as a method to regenerate catalysts that have been affected by coking in methanation reactors.^11^

The effect of coking on a catalyst can be quantified either by the amount of carbon deposits,^12,13^ or by the change of catalyst activity,^12,14^ defined as a percentual decrease of the reaction rate on a catalyst in time as an effect of any deactivation mechanism. The latter is more helpful in the modeling of reactors whose main goal is other than the production of solid carbonaceous structures. The concentration of coke can be related to the activity of the catalyst, and so can the rate of its formation and the rate of catalyst deactivation. Several examples of deactivation kinetics are available in the literature.^13–15^ A common approach is to define rate equations for coke deposition or for the decrease of catalyst activity. The dependence of the rate equations on the local operating conditions is modeled via simplified kinetic expressions^16^ or via a mechanistic approach based on quasi-stationary consecutive reaction steps on the catalyst.^17,18^ While the latter approach is rigorous, the former is mathematically simpler and thus more useful for practical applications. In fact, this approach was proven to accurately represent most sets of experimental data.^13^

While some kinetic models for deactivation by coking have been developed in the literature, the examples of deactivation models at the reactor scale are scarce and lack generality. Examples of deactivation models in reactors include the work by Chen and Weng on well-mixed reactors,^19^ while details on the deactivation at the active-site and particle level, as well as a simple framework for its implementation on a small-scale reactor model, were provided by Froment.^20^ Cordero-Lanzac et al.^21,22^ modeled the fast catalyst deactivation in fixed-bed as well as bubbling and circulating fluidized-bed reactors by including catalyst deactivation rates of similar orders of magnitude to the reaction kinetics in a parallel compartment model. It is, however, still a challenge to build a model characterized by sufficient computational efficiency to allow for reactor optimization, regardless of the time scale of the deactivation. Such a time scale has in fact a strong influence on the numerical stiffness of the model and therefore on its computational efficiency. The analysis of reactor performance over large time scales is relevant for the catalytic processes in the field of low-carbon technologies, which show slow deactivation causing loss of performance over long times on stream. It is therefore crucial to develop standardized methods for the assessment and minimization of the impact of coking on this class of catalytic reactors. Specifically in the field of CO_*x*_ conversion, the availability of such methods would greatly help to make these processes more competitive and efficient in the long run.

As the coking phenomenon itself is dependent on the operating conditions, the optimization method must allow for the individuation and selection of the optimal values of such conditions, meaning those that allow for a minimum loss of conversion over the time on stream. The lack of such a method is a major problem for the development of low-carbon technologies, as many reactions involved in the production of fuels from sustainable sources are carried out in fluidized-bed reactors.^23^ This class of reactors is especially useful when dealing with exothermicity. In fact, the virtually instant mixing effect provided by the circular movement of the catalyst particles (and their thermal inertia) in this reactor configuration is responsible for a homogeneous temperature profile, as opposed to fixed-bed reactors, whose operation with exothermic reactions is characterized by the presence of a hotspot. The absence of a hotspot in fluidized-bed systems leads to a much lower thermal stress on the catalyst and minimizes the generation of side products by side reactions happening at high temperatures.^24^ Besides, catalyst deactivation by carbon deposition is hindered by the constant recirculation of the catalyst particles between an initial reactant-rich zone of the reactor, in which carbon deposition happens, and a final product-rich zone. The main difficulty related to the use of fluidized-bed reactors in such system is related to the lack of a tool for systematic *operando* study of catalytic surfaces of moving particles: the main methods for *operando* investigation of deactivation are in fact related to fixed-bed technology.^25^

The assessment of deactivation in fixed-bed reactors is mainly carried out by Raman spectroscopy, both ex situ^26–29^ and *in operando*.^30–33^ Some deactivation studies deal with CO_*x*_ hydrogenation like this work.^26,34–36^ However, a comprehensive study of catalyst deactivation in CO_*x*_ hydrogenation, combined with the consequent reactor performance loss, is lacking. For fluidized-bed reactors, *operando* spectroscopy techniques could not be combined with the chaotic movement of the particles so far, while ex situ studies are still possible. Even in this case, no literature is available on the characterization of the deactivation process and reactor optimization.

The goal of this study is to develop a methodology that can group together in a single model information relating to all spatial and temporal scales of deactivation. The smallest scale involved is that of the single particle, where coke deposition happens, leading to a loss of active surface. The largest scale is that of the reactor, whose ability to convert carbon into fuels decreases over time on stream as an effect of the loss of catalyst activity. The model must achieve computational affordability, i.e., provide useful results in a practical time span. The model developed in this work can collect three levels of information, delegated to three dedicated submodels. One level is the instant composition profile inside the reactor, which is calculated assuming a steady state due to the much shorter time scale compared to that of catalyst deactivation. Another submodel, exclusive to fluidized beds, calculates the instant position of an average particle, representative of all the catalysts in the reactor. Finally, a submodel dedicated to deactivation kinetics uses the information on composition profiles and particle position to calculate the instant rate of deactivation of the catalyst, either at each position of the bed in the case of fixed beds or for a single representative particle in the case of fluidized beds. The rate of activity loss is then integrated over the time on stream to move to a subsequent instant, in which the activity is lower than before, and a new steady-state operation (slightly different than the previous one) is achieved in the reactor. This methodology has been named *Pseudodynamic model* in the case of fixed-bed reactors, and *Moving Observer model* for fluidized beds.

This review is structured in different sections pertaining to different stages of the work. The Methodssection will be initially focused on the global architecture of the *Pseudodynamic* and *Moving Observer* models, and on illustrating the interplay between the different submodels that constitute it. Consequently, a subsection will be dedicated to explaining each of the different submodels. For each submodel, the development process will be explained thoroughly from its theoretical bases to its final characteristics. The Results and Discussionsection will initially illustrate the validation of the submodels. Afterward, the main outcomes of the simulation of a pilot-scale reactor over a long time on stream will be explained. The loss of catalyst activity in the fixed bed and the subsequent decrease of conversion of the main reactant will be calculated over up to 500 h of operation for four equally spaced inlet temperatures between 300 and 360 °C. For the fluidized bed, the same temperature values will be investigated as uniform bed temperatures, and the calculations will continue up to 2000 h of operation. In the Conclusionssection, the main takeaways of the study will be illustrated, as well as the options for future developments and improvements.

## Methods

This work aims to develop a model to assess the impact of catalyst deactivation on the efficiency and performance of a chemical reactor. The model developed here is based on the decoupling of the temporal scales of the different phenomena happening in the reactor. Indeed, the reaction and transport phenomena in a chemical reactor happen on a time scale of seconds or lower, the particle motion (only for fluidized systems) on a scale of minutes, and the deactivation on a scale of hours or higher. This allows us to consider the operation of the reactor along the time on stream as characterized by a succession of steady states at different values of the catalyst activity with respect to reaction *j*, which is defined in eq 1. In this definition, *r*_*j*_ is the reaction rate of reaction *j* at any set of conditions and any deactivation extent, while *r*_*j*_^0^ is the rate of the same reaction, happening at the same conditions but on a fresh catalyst.

1 $$a_{j}=\frac{r_{j}}{r_{j}^{0}}$$

By assuming that the reactor is operating according to a sequence of steady states, we are decoupling the time scale of the deactivation from that of the other phenomena happening in the reactor. The resulting model will therefore be constituted by a single ordinary differential equation (ODE) per reaction in the variable activity *a*_*j*_, and by a set of submodels that will be called at each integration step and are listed below. The model ODE is expressed in eq 2

2 $$\frac{da_{j}}{dt}=-r_{d,j}a_{j}$$

1. A steady-state reactor model will calculate the composition profiles *x* = *f*(*z*) in the reactor at any given time *t* and value of *a*_*j*_, by assuming the reactor is operating at the steady state.
2. A deactivation kinetic model will calculate the rate *r*_*d*_ at which the catalyst deactivates from the composition around it, determined by the position along the axial reactor coordinate *z*.
3. For the fluidized-bed case only, a particle motion model will calculate the position of a single particle chosen as representative of the average particle in the bed. Due to the assumption of perfect mixing of the catalyst in a fluidized-bed reactor, the activity of that same particle will be used to calculate the reaction rates throughout the whole bed volume.

For the fixed bed instead, the activity will not be a single value but a profile along *z*, and the instant decrease of activity will be also calculated as a profile according to the composition profile

3 $$r_{d}^{\mathrm{fix}}=f\left(\mathrm{p̲}\left(z\right)\right)=f\left(z\right)$$

A schematic representation of the model architecture and of the roles of the submodels is shown Figure 1. The deactivation model for the fixed-bed reactor has been labeled *Pseudodynamic model* as it achieves a dynamic modeling of the reactor without the use of partial differential equations. The fluidized-bed reactor deactivation model has instead been named the *Moving Observer model*, as it follows the motion of an average particle throughout the bed.

**Figure 1:**
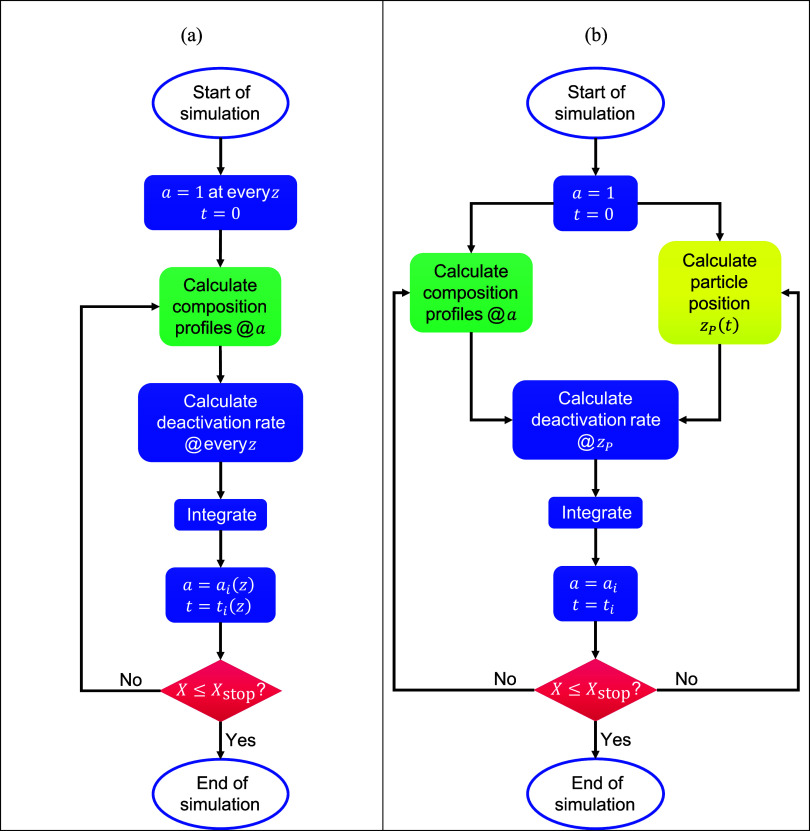
Model structure for the *Pseudodynamic model* (fixed bed, a) and *Moving Observer model* (fluidized bed, b). *X* indicates the conversion of methane at the outlet of the reactor. Boxes in green refer to the reactor submodels, and boxes in yellow refer to the particle tracking submodel.

The equations are solved in MATLAB through the built-in integrator for stiff problems, *ode15s*.

### Case Study and Kinetics

For both reactor types, the corresponding submodel is based on an existing pilot-scale reactor and has been validated with data from CO_2_ methanation due to higher data availability.^37,38^ However, as the methanation of CO_2_ shows a slower deactivation than the methanation of CO,^39^ the latter has been used as a case for this study. The fixed-bed reactor is a plate-cooled type, with rectangular channels alternatively used for reaction and cooling. The reactor is 1 m long, 50 cm wide, and has 5 cm of spacing between plates;^37^ it is operated with a stoichiometric inlet for CO methanation (3:1 H_2_/CO), at a pressure of 8 bar and with an inlet temperature of 300, 320, 340, or 360 °C. The fluidized-bed pilot reactor is characterized by a bed height of 2.5 m, a diameter of 22.4 cm, and operated at the same conditions as the fixed bed and isothermally.^40^

The kinetics used are derived from Koschany et al.^41^ (CO_2_ methanation reaction) and Kopyscinski et al.^42^ (CO methanation and water–gas shift reactions), as per Table 1. The stoichiometric effect of the decomposition of CO to coke (assumed as elemental carbon, C) and CO_2_ according to the Boudouard reaction^43^ is not considered in the kinetic equations.

**Table 1:** Reactions in the Kinetic Submodel for CO Methanation^41,42^

*j*	reaction	equation
1	CO_2_ methanation	${\mathrm{CO}}_{2}+4H_{2}⇄{\mathrm{CH}}_{4}+2H_{2}O$
2	water–gas shift	$\mathrm{CO}+H_{2}O⇄{\mathrm{CO}}_{2}+H_{2}$
3	CO methanation	$\mathrm{CO}+3H_{2}⇄{\mathrm{CH}}_{4}+H_{2}O$

The reaction rates in $\left[\frac{\mathrm{kmol}}{s\cdot {\mathrm{kg}}_{c}}\right]$ are expressed by the following equations

4 $$r_{1}=\frac{k_{1}p_{H_{2}}^{0.5}p_{CO_{2}}^{0.5}\left(1-\frac{1}{K_{1}^{eq}}\frac{p_{CH_{4}}p_{H_{2}O}^{0.5}}{p_{CO_{2}}p_{H_{2}}^{4}}\right)}{{\left(1+K_{\mathrm{OH},1}\frac{p_{H_{2}O}}{p_{H_{2}}^{0.5}}+\sqrt{K_{H_{2}}p_{H_{2}}}+K_{mix}p_{CO_{2}}^{0.5}\right)}^{2}}$$

5 $$r_{2}=\frac{k_{2}\left(K_{\alpha }p_{\mathrm{CO}}p_{H_{2}O}-\frac{1}{K_{2}^{\mathrm{eq}}}p_{H_{2}}p_{CO_{2}}\right)}{{\left(1+K_{C}p_{\mathrm{CO}}^{0.5}+K_{\mathrm{OH},2}\frac{p_{H_{2}O}}{p_{H_{2}}^{0.5}}\right)}^{2}}$$

6 $$r_{3}=\frac{k_{3}\left(K_{C}p_{\mathrm{CO}}^{0.5}p_{H_{2}}^{0.5}-\frac{1}{K_{3}^{\mathrm{eq}}}p_{{\mathrm{CH}}_{4}}p_{H_{2}O}\right)}{{\left(1+K_{C}p_{\mathrm{CO}}^{0.5}+K_{\mathrm{OH},2}\frac{p_{H_{2}O}}{p_{H_{2}}^{0.5}}\right)}^{2}}$$

For all reactions, the kinetic constant is obtained through a modified Arrhenius equation

7 $$k_{j}=k_{j}^{\mathrm{ref}}\;\exp\left(\frac{E_{j}}{R}(\frac{1}{T_{\mathrm{ref}}}-\frac{1}{T})\right)$$

where the reference temperature is 555 K for the first reaction and 598.15 K for the remaining two. Similarly, the adsorption constants *K*_*k*_ behave according to eq 8

8 $$K_{k}=K_{k}^{\mathrm{ref}}\;\exp\left(\frac{{\Delta h}_{k}^{\mathrm{ads}}}{R}(\frac{1}{T_{\mathrm{ref}}}-\frac{1}{T})\right)$$

with the same reference temperatures as before. The index *k* refers to each single adsorbate involved in the kinetic equations above.

The parameters for the kinetic submodel are reported in the Supporting Information.

The equilibrium constants are obtained from the following relations

9 $$K_{1}^{\mathrm{eq}}=137\cdot T^{-3.998}\cdot \exp\left(\frac{1.587\cdot 10^{5}}{\mathrm{RT}}\right)$$

10 $$K_{2}^{\mathrm{eq}}=10^{\left(2.4198+3.855\cdot 10^{-4}T+\left(2180/T\right)\right)}$$

11 $$K_{3}^{\mathrm{eq}}=10^{\left(-30.42+\left(27106/T\right)\right)}$$

### Fixed-Bed Reactor Submodel

The fixed-bed steady-state submodel is of the one-dimensional (1D) pseudohomogeneous type.^44^ No transport limitation is assumed to exist between the gas and the catalyst and within each of the two phases, and gradients of composition and temperature are also neglected in any direction other than the gas flow. The gas mixture is assumed to behave as a plug flow. Equtions [Disp-formula eq12] and 13 are the component and energy balances for the reactor, respectively

12 $$\frac{dF_{i}}{dz}=\rho_{\mathrm{pb}}\left(1-\varepsilon \right)A\sum_{j=1}^{\mathrm{NR}}a_{j}\left(t\right)\nu_{\mathrm{ij}}r_{j}^{0}$$

13 $$\frac{dT}{dz}=-\frac{\rho_{\mathrm{pb}}\left(1-\varepsilon \right)A}{ṁ{ĉ}_{P}}\sum_{j=1}^{\mathrm{NR}}a_{j}\left(t\right)\Delta h_{j}^{R}r_{j}-\frac{2W}{ṁ{ĉ}_{P}}U\left(T-T_{e}\right)$$

The resulting submodel is an ODE system of *NC* + 1 equations in *z*, with *NC* being the number of chemical species involved. Like the global model, it is solved with the built-in MATLAB integrator for stiff problems *ode15s*.

### Fluidized-Bed Reactor Submodel

The fluidized-bed steady-state submodel is based on a 2-phase assumption.^45–47^ The reactor is thus divided into two phases:

1. a bubble phase, assumed to contain no catalyst (thus nonreactive) and to behave as a plug flow,
2. a dense or emulsion phase, assumed to be reactive and perfectly mixed.

In the following, when talking about any local value of composition in the fluidized-bed reactor model, we will refer to a weighted average of the two phases unless otherwise specified, as some amount of gas mixture is always present in both phases during the operation of the reactor and the outlet composition is anyway a result of this. The perfect mixing assumption for the solid in the emulsion is reasonable due to the fast movements of the bubbles in the reactor. It holds under the assumption of uniform size and density of the particles when the gravitational, buoyancy, and attrition forces acting on each particle can safely be assumed equal. A nonuniform particle size distribution or the presence of particles of different nature in the same fluidized bed might influence the mixing in the reactor.^48,49^ However, this effect is not interesting in the scope of this work. This assumption also leads to a uniform temperature profile in the reactor, therefore to an isothermal submodel expressed by equations 14 and 15

14 $$\frac{dF_{i}^{B}}{dz}=-\Gamma_{i}A_{B}\left(\frac{F_{i}^{B}}{Q_{B}}-\frac{F_{i}^{D}}{Q_{D}}\right)$$

15 $$0=F_{i}^{D,\mathrm{IN}}-F_{i}^{D}+\int_{0}^{L}\Gamma_{i}A_{B}\left(\frac{F_{i}^{B}}{Q_{B}}-\frac{F_{i}^{D}}{Q_{D}}\right)dz+V\left(1-\delta \right)\left(1-\varepsilon_{\mathrm{mf}}\right)\rho_{c}\sum_{j=1}^{\mathrm{NR}}a_{j}\left(t\right)\nu_{\mathrm{ij}}r_{j}^{0}$$

The correlations to calculate the fluidization parameters *A*_*B*_, ε_mf_, *Q*_*B*_, and *Q*_*D*_ are taken from the work of Abashar and Al-Rabiah^24,45,50^ as they fit the range of operating conditions treated in this work. They are shown together in Table 2.

**Table 2:** Fluidization Correlations

16 $$\mathrm{Ar}=\frac{d_{p}^{3}\rho_{G}g\left(\rho_{C}-\rho_{G}\right)}{\mu^{2}}$$
17 $$\varepsilon_{\mathrm{mf}}=0.586{\mathrm{Ar}}^{0.029}{\left(\frac{\rho_{G}}{\rho_{C}}\right)}^{0.021}$$
18 $$u_{\mathrm{mf}}=\frac{\mu }{\rho_{G}d_{P}}\left(\sqrt{25.25^{2}+0.0651\mathrm{Ar}}-25.25\right)$$
19 $$Q=u_{g}^{0}A$$
20 $$Q_{D}=u_{\mathrm{mf}}A$$
21 $$Q_{B}=Q-Q_{D}$$
22 $$d_{\mathrm{Bm}}=0.652{\left(A\left(u_{G}^{0}-u_{\mathrm{mf}}\right)\right)}^{0.4}$$
23 $$d_{B}^{0}=0.347{(7.85\cdot 10^{-5}\left(u_{G}^{0}-u_{\mathrm{mf}}\right)}^{0.4}$$
24 $$d_{B}=d_{B}^{m}-\left(d_{B}^{m}-d_{B}^{0}\right)\;\exp\left(-0.3\frac{z}{D_{R}}\right)$$
25 $$A_{B}=\frac{\pi }{4}d_{B}^{2}$$
26 $$u_{B}=u_{G}^{0}-u_{\mathrm{mf}}+0.711{\left(gd_{B}\right)}^{0.5}$$
27 $$\delta =\frac{u_{G}^{0}-u_{\mathrm{mf}}}{u_{B}}$$
28 $$H=\frac{H_{\mathrm{mf}}}{1-\delta }$$
29 $$D_{\mathrm{ij}}=\frac{0.04357T^{3/2}\sqrt{\frac{1}{M_{i}}+\frac{1}{M_{j}}}}{P{\left({v_{i}^{c}}^{1/3}+{v_{j}^{c}}^{1/3}\right)}^{2}}$$
30 $$D_{i}^{m}=\frac{1-x_{i}}{\sum_{j=1,j\neq i}^{\mathrm{NC}}\frac{x_{i}}{D_{\mathrm{ij}}}}$$
31 $$\Gamma_{i}^{\mathrm{BC}}=4.5\frac{u_{\mathrm{mf}}}{d_{b}}+5.85{D_{i}^{m}}^{1/2}g^{1/4}d_{B}^{5/4}$$
32 $$\Gamma_{i}^{\mathrm{CD}}=6.78\sqrt{\varepsilon_{\mathrm{mf}}D_{i}^{m}}$$
33 $$\Gamma_{i}={\left(\frac{1}{\Gamma_{i}^{\mathrm{BC}}}+\frac{1}{\Gamma_{i}^{\mathrm{CD}}}\right)}^{-1}$$

The model is of the differential-algebraic-integral type (*DAIE*), thus inherently stiff and unsolvable by an explicit integration algorithm like *ode15s*. Even though the solver *ode15s* is capable of solving DAE problems through a mass matrix, the presence of an integral term (hence DAIE) in the species balance in the dense phase makes it necessary to use a finite-difference discretization, as the integral term must be calculated considering the whole flow rate profiles at the same time, and it influences the profiles themselves. Hence, this requires the definition of a numerical integration routine. The discretization of the spatial derivative through a central, second-order differentiation scheme (eq 26) and the calculation of the integral through a trapezoidal rule allow for its transformation in a set of algebraic equations which can be solved through the built-in *fsolve* function in MATLAB.

34 $${\frac{dy}{dz}|}_{z_{k}}=\frac{y_{k+1}-2y_{k}+y_{k-1}}{z_{k+1}-z_{k-1}}$$

### Particle Motion Submodel

A model for particle motion has been derived from the experimental data obtained by Lefebvre et al.,^51–53^ normalized on the dimensions of the reactor studied in this work. The experimental data are shown in Figure 2 together with the model. One can observe that the probability that a particle stays in a section of the reactor is not the same for every height of the reactor itself. In fact, it was described in the literature that the size of the bubbles is different at various heights of a bubbling fluidized-bed reactor.^40^ Where the bubbles are big and fast (i.e., on the top of the reactor), the residence time of a particle is high, as demonstrated in Figure 2, where the residence time at a high level is higher than at a low level. However, one should consider that the accuracy of this description has a low impact on the overall validity of the methodology, as the most relevant driving force for deactivation is the residence time of the particle in carbon-rich areas of the reactor.

**Figure 2:**
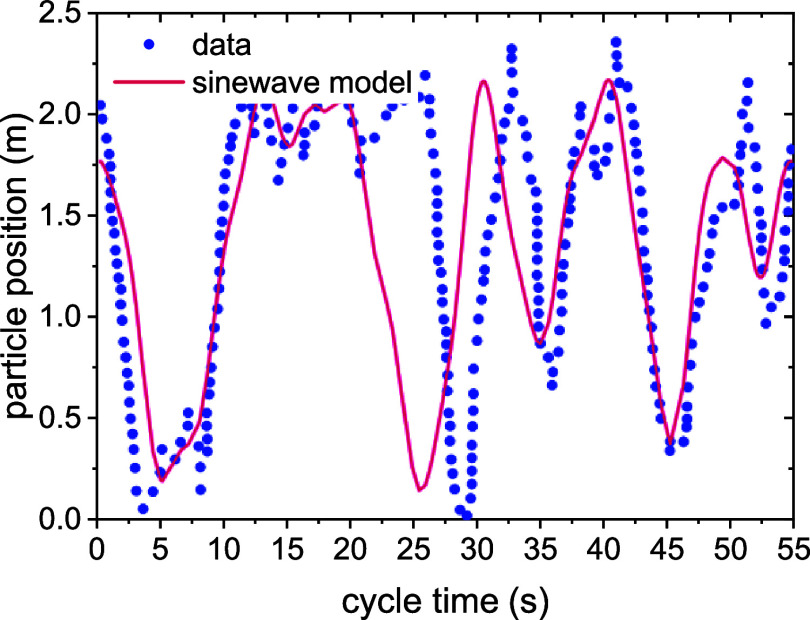
Particle tracking data from refs 51–53 and sinewave model adapted to them.

The data have been analyzed through a discrete Fourier transform to obtain a wave function that fits the trends observed in the data and conserves its periodicity. Wave components with a negligible impact on the particle position have been filtered out to make the resulting model simpler (labeled as *Sinewave model*). The resulting fit is shown in Figure 2. The model aims to define a certain periodicity in the particle movement rather than describing the particle movement itself accurately. A precise description of the particle movement is not needed due to the different time scales between the movements of the particles and deactivation.

One should note that even though the function does not superimpose perfectly with the data, it attains two goals: it represents the overall time spent by the particle in each portion of the reactor and it is mathematically simple and periodic. Other, more complex model functions have been discarded in the process due to their higher mathematical complexity.

### Deactivation Submodel

Many different kinetic models for catalyst deactivation are available in the literature.^13^ They aim to characterize the evolution of either the concentration of coke on a particle or the activity in time. The activity expresses the reaction rate on a catalyst particle under given conditions as a fraction of the reaction rate under the same conditions on the fresh catalyst. A kinetic expression for the concentration of coke has the form expressed in eq 27, while the one for the activity is shown in eq 28

35 $$\frac{dc}{dt}=f_{c}\left(\mathrm{x̲},T,c\right)$$

36 $$\frac{da}{dt}=f_{a}\left(\mathrm{x̲},T,a\right)$$

Two options are available for the determination of the dependence of *f*_*c*_ or *f*_*a*_ on the operating conditions and current values of the coke concentration and activity. A more simplified one relies on the use of power-law-like expressions of the kind shown in eq 29 ^16,54^

37 $$f_{a}\left(\mathrm{x̲},T,a\right)=-k_{d}\prod_{i}x_{i}^{\alpha_{i}}a^{\beta }$$

A more rigorous approach is based on the derivation of a quasi-stationary stepwise kinetic mechanism of the deposition of coke on the active sites of a catalytic particle.^17,18^ Such expressions are usually more complex and rely on calculations of coverage fractions for the species involved in the reaction, often without a significant increase in their ability to fit experimental data. Consequently, a model of the power-law type was chosen for this study. It is based on an adaptation and reparameterization of a model by Sun et al.^14^ The resulting submodel is based on the equations listed in Table 3

**Table 3:** Equations of the Deactivation Kinetics

name	equation
deactivation rate (main model equations)	38 $\frac{da_{j}}{dt}=-r_{d,j}a_{j}$
kinetic equation	39 $r_{d,j}=k_{d,j}p_{\mathrm{CO}}^{\alpha }f_{H_{2}O}$
temperature dependence (Arrhenius)	40 $k_{d,j}=k_{d,j}^{0}\;\exp\left(-\frac{E_{d,j}}{\mathrm{RT}}\right)$
correction factor for water	41 $f_{H_{2}O}=\frac{x_{H_{2}O}-1}{\left(1-c\right)x_{H_{2}O}-1}$

The correction factor in eq 32 is an empirical way to account for the hindering effect of water on deactivation phenomena. It is not, however, a measure of the regeneration effect that water could operate on the catalyst by gasifying the coke. This effect is in fact not relevant to our study, as it has been shown to happen only starting from 400 °C.^11^ These conditions are never met in the fluidized bed in the cases simulated in this work and are only reached in the fixed bed when and where the catalyst is still active, so no regeneration effect can be present.^14^ The dependence of the correction factor on the partial pressure of water is shown in Figure 3. The effect of water on deactivation will be studied in more detail in future works.

**Figure 3:**
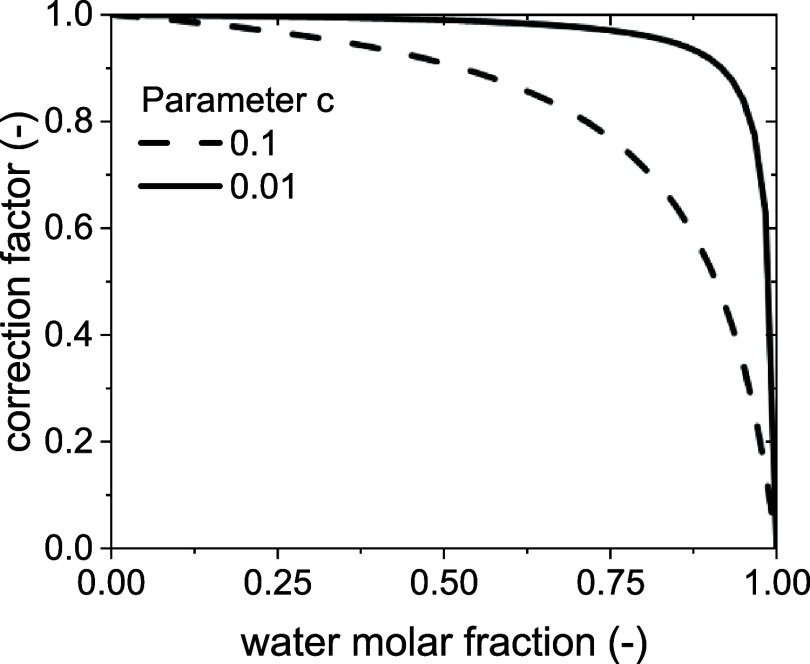
Effect of the water content on the correction factor.

The other model parameters were obtained through a parametrization according to the catalyst deactivation data reported by Zhang et al.^55^ The data are based on a case study on CO methanation in a lab-scale reactor at five temperatures between 300 and 360 °C, of which the three values of 320, 340, and 360 °C were selected. The original data showed the evolution of the conversion at the reactor outlet over time on stream. For simplicity, the reparameterization done in this study was based on a simplified definition of the activity as the ratio between the overall conversion in the reactor and its initial value, i.e., its value at zero time on stream

42 $$a_{r}=\frac{X}{X_{0}}$$

This alternative definition of activity is indicated with *a*_*r*_ to differentiate it from the more rigorous one outlined in eq 1.^47^

## Results and Discussion

### Validation of the Fixed-Bed Reactor Submodel

The comparison between a temperature profile obtained from the steady-state reactor submodel and experimental data from a pilot-scale reactor of the same dimensions and at the same conditions^37^ is reported in Figure 4.

**Figure 4:**
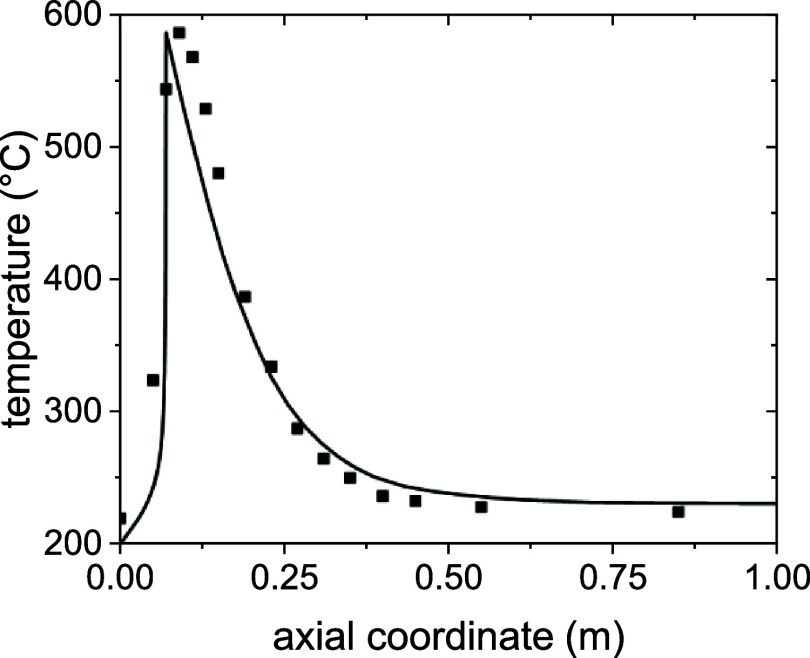
Validation of the steady-state fixed-bed reactor submodel with data from ref 37.^37^

The study by Moioli et al. reports a case of biogas upgrading by methanation of CO_2_, with no CO in the feed, on a catalyst that has been shown to be perfectly selective toward CH_4_. This implies that the conversion and temperature profiles are univocally dependent; thus, the good agreement between the calculated temperature profile and experimental points shown in Figure 4 is enough to validate the model. The slight disagreement between the two profiles is attributed to the difficulty of the model to predict the effect of the metallic structure of the reactor in transporting the heat, making the temperature profiles more uniform along the axial direction.

### Validation of the Fluidized-Bed Reactor Submodel

The comparison of simulation data for the dry composition profiles of H_2_, CH_4_, and CO_2_ with the corresponding experimental data from a fluidized-bed, pilot-scale reactor^38^ is shown in Figure 5.

**Figure 5:**
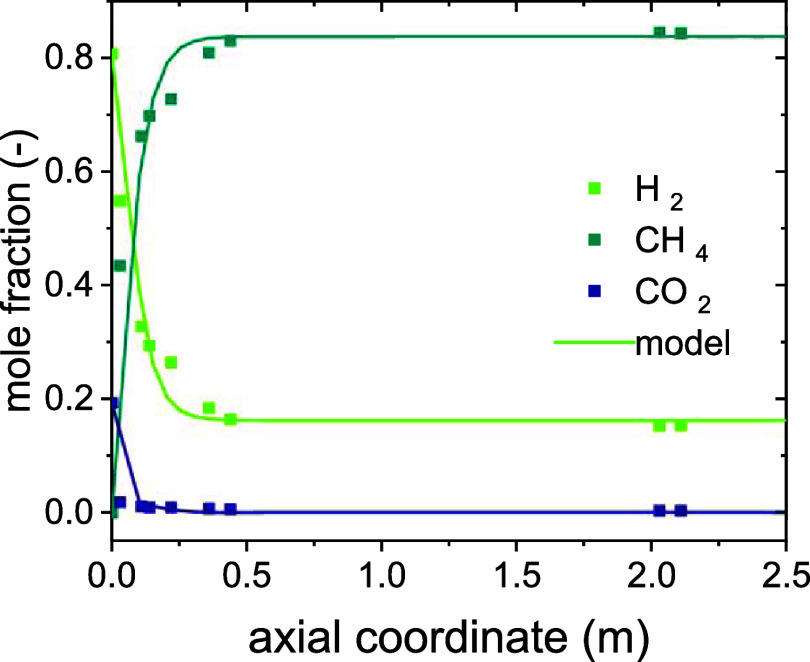
Validation of the fluidized-bed reactor submodel at 300 °C, 8 bar, and a GHSV of 1000 h^–1^.

The figure shows that the submodel of the pilot-scale reactor can accurately predict the steady-state composition profiles of an experimental run at the same conditions. The accuracy of the predictions by the submodel allows for its use in the *Moving Observer model*.

### Deactivation Submodel

A summary of the final values used for the deactivation submodel parameters, obtained from the fitting of data from Zhang et al.,^55^ is reported in Table 4. As CO methanation can be assumed to be perfectly selective to methane, a single definition of activity was used. The parameters were determined by regression over all of the available experimental data.

**Table 4:** Final Deactivation Submodel Parameters

symbol	parameter	value	unit
*k*_*d*_^0^	preexponential factor	0.182	-
*E*_*d*_	activation energy	48,418	$\frac{J}{\mathrm{mol}}$
α	reaction order (CO)	1	-
*c*	correction intensity	0.01	-
β	activity influence parameter	1	-

The values of the CO reaction order and correction intensity are preliminary and have been determined arbitrarily. Further experimental analyses will be carried out in the future. The fit with the data resulting from the parameters in Table 4 is shown in Figure 6.

**Figure 6:**
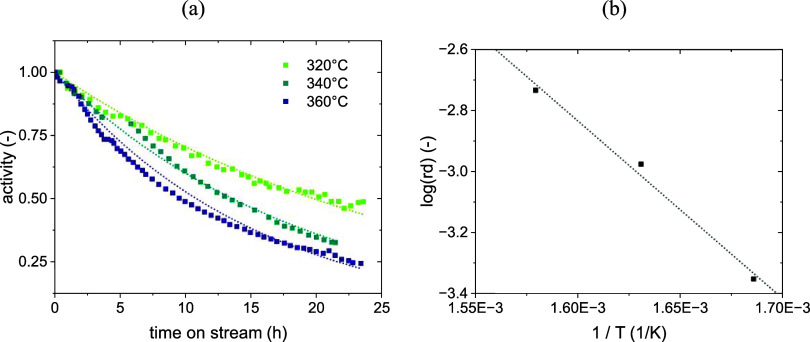
Results of the regressions used to calibrate the deactivation submodel (experimental data from ref 32, definition of activity from eq 33 (a) and Arrhenius plot of the deactivation rate constant (b)).

The model shows an ability to describe the evolution of the activity over time on stream—calculated according to eq 33—as an effect of deactivation. The deactivation proceeds faster at a higher temperature, due to the faster decomposition of the carbon-containing species at a higher temperature according to the Boudouard equilibrium.^43^ The apparent preexponential factor and activation energy for the deactivation have been calculated by comparing the deactivation rates at different temperatures, as shown in Figure 6b.

### Pseudodynamic Model

The *Pseudodynamic model* has been used to evaluate the loss of CO conversion due to methanation in a fixed-bed reactor with the catalyst subject to deactivation. The reactor is operated under the following conditions: 50 cm channel width, 1.5 cm channel height, 1 m channel length, and a gas hourly space velocity (GHSV) of 500 h^–1^. The simulation has been carried out at four different inlet temperature values (300, 320, 340, 360 °C), and for a maximum time on stream of 100 h, although an early stop to the run was introduced whenever the conversion of CO at the outlet of the reactor dropped below 25%, at which point the reactor was considered to not perform anymore. As a benchmark for the coming discussion, the CO conversion and temperature profiles for the fresh catalyst (*a* = 1 in the whole reactor) at an inlet temperature between 300 and 360 °C are shown in Figure 7.

**Figure 7:**
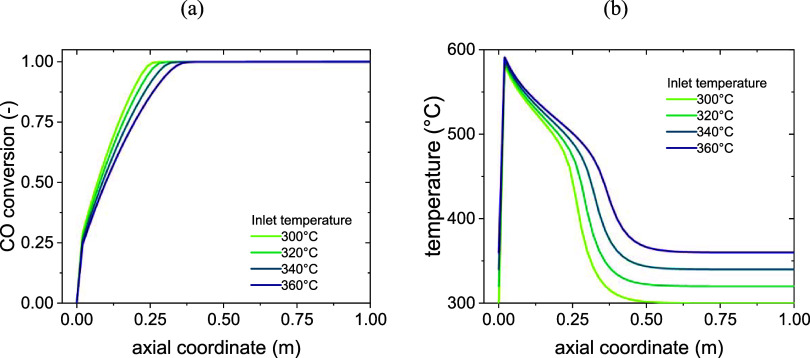
Conversion (a) and temperature profiles (b) for CO methanation on fresh catalyst according to the fixed-bed submodel.

Three sections can be identified.

- The first 5÷10 cm can be called a *kinetic zone*. It is characterized by a high concentration of reactants, leading to a high reaction rate, a rapid increase of conversion, and a consequent fast release of heat. This leads to the generation of a hotspot, with temperatures almost up to 600 °C. The conversion reaches 30% in this section of the reactor.
- The following 15÷20 cm can be labeled as *heat-transfer-controlled zone*. The depletion of the concentration of reactants in the kinetic zone leads to a slower reaction rate and release of heat in this section. The influence of the cooling system brings the temperature down, but the reaction keeps proceeding, though at a slower rate. The conversion keeps increasing, though slowly, until the thermodynamic limit is reached, which in these conditions corresponds to almost 100% conversion.
- The remainder of the reactor can be considered a *thermodynamic zone*, in which no reaction happens due to the thermodynamic limit.

The evolution of these profiles due to catalyst deactivation according to the model is depicted in Figures 8b and[Fig fig9]a, while Figure 8a shows the activity profile, initially identically equal to 1. At the very start, only the kinetic zone seems to be affected by deactivation. In this section, the activity rapidly drops and so does the conversion of CO. This is mainly due to the hotspot being localized here: the rate of deactivation of the catalyst is in fact boosted by the high temperature, which leads to a faster local deactivation than that in the rest of the reactor. The initial kinetic zone turns in an *inactive zone*, in which no reaction happens due to the deactivation of the catalyst. At the same time, the following 5÷10 cm turn into a new kinetic zone, as the concentration of reactants is still high, and the catalyst has not lost a significant amount of activity yet. The hotspot also moves to this new kinetic region. While the kinetic zone moves forward in the reactor, so do the heat transfer and thermodynamic ones.

**Figure 8:**
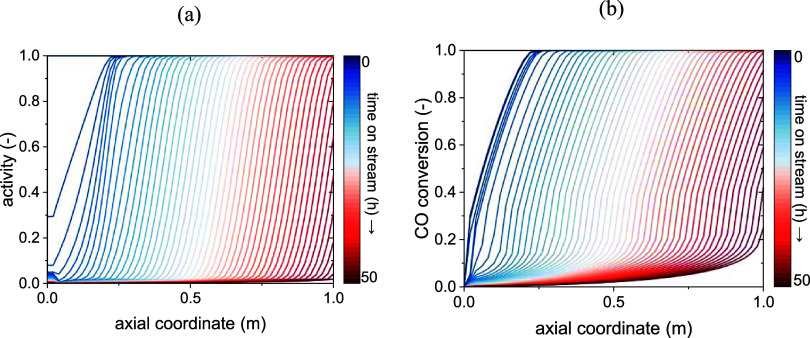
Results of the *Pseudodynamic model*: deactivation wave (a) and resulting conversion profile (b) at 300 °C inlet temperature (activity defined as per eq 1).

**Figure 9:**
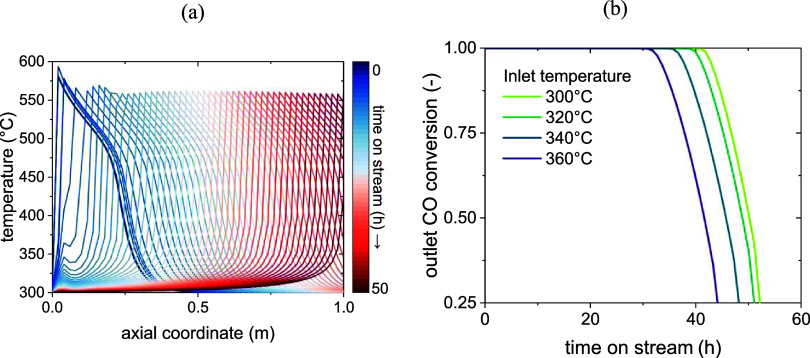
Moving hotspot at 300 °C inlet temperature (a) and effect of temperature on deactivation (b) according to the model.

While the inactive zone expands due to deactivation, the others move along the axial reactor coordinate, forming a deactivation front. It can be noted from Figure 9b that the conversion at the outlet of the reactor is not affected until the heat transfer zone has reached the outlet, since the thermodynamic region is characterized by the maximum conversion value in the reactor. This happens after about 40 h, after which the outlet conversion starts to decrease until the limit of 25% set by our simulation.

Figure 9b also compares the effects of different inlet temperatures on the deactivation. A higher inlet temperature leads to a faster kinetics at the inlet, higher temperatures throughout the reactor, and therefore to overall faster deactivation according to the deactivation kinetic submodel. At 360 °C, the deactivation front takes about 10 h less to reach the outlet of the reactor than at 300 °C. The influence of the temperature profile is hence extremely impactful on the catalyst deactivation, so that the heat management is of paramount importance. The sensitivity to the heat transfer coefficient was tested at 300 °C, resulting in almost 80 h prior to deactivation for a 2-fold increase in the heat transfer and 171 h for a 5-fold increase, compared to the 45 h needed with the heat transfer calculated from correlations. The corresponding evolution of the outlet conversion is reported in the Supporting Information, as well as the temperature profiles for the global heat exchange coefficient calculated by the model and for a 5-fold increase.

Apart from temperature, several other parameters have an influence on catalyst deactivation. For example, a higher amount of CO would significantly decrease the time on stream as the fraction of the reactor with the presence of CO would increase. Higher flow rates (or GHSV) would also impact the deactivation extent due to the delayed conversion of CO with the axial coordinate.

### Fluidized-Bed Reactor Model According to the *Moving Observer* Concept

Like in the *Pseudodynamic* case, the *Moving Observer model* was employed in the calculation of the CO conversion in the methanation reaction over time on stream in a fluidized-bed reactor subject to deactivation. The reactor is operated under the following conditions: 22.4 cm reactor diameter, 2.5 m bed height, and 1000 h^–1^ gas hourly space velocity. Four different reactor temperatures have been simulated (300, 320, 340, 360 °C) for a maximum time on stream of 2000 h, with an early stop for conversions equal to or below 25%. The fresh-catalyst conversion profile for a reactor temperature of 300 °C is shown in Figure 10.

**Figure 10:**
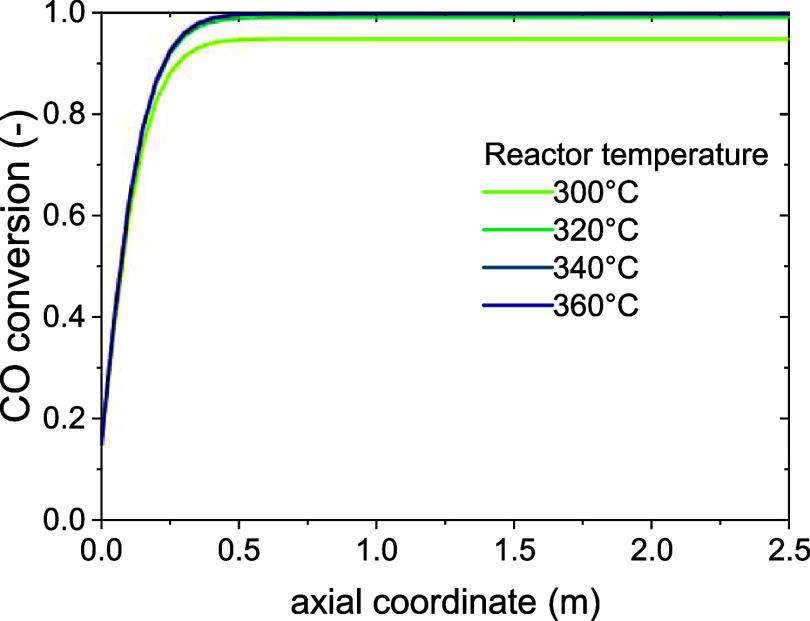
CO conversion profiles on the fresh catalyst according to the fluidized-bed model.

It must be noted that the composition in the reactor is calculated as a weighted average of the compositions of the two phases, as explained in the Methodssection, hence the nonzero conversion at the reactor inlet. Different from the fixed-bed profiles shown in Figure 7, the fluidized-bed conversion profile shows a steady increase in the conversion up to the thermodynamic limit. This is an effect of the recirculation of the emulsion (dense phase, mixture of catalyst and surrounding gas) operated by the bubbles, which makes the composition of the gas in the emulsion, and so the reaction rate, uniform throughout the reactor.

Two zones can be identified for simplicity: a *reactive zone* in which the conversion increases along *z*, and a *thermodynamic zone* in which the thermodynamic limit has been reached and the reaction is at equilibrium. The former is rich in CO (and hydrogen), and the other is rich in water (and methane).

Figure 11 shows the loss of conversion in the reactor over time on stream at the same temperature. It is evident that, differently from the fixed-bed case, the conversion drops uniformly throughout the bed as the catalyst deactivates, regardless of zones. This is also an effect of the assumption of a perfect mixing in the dense phase, where the reaction happens, and the catalyst is located. As the dense phase composition is considered uniform along the axial coordinate of the reactor, so is the rate of deactivation on the catalyst. This is another reason why, for this model, the activity has been modeled on a single particle representative of the whole fluidized bed. As a result, the conversion at the reactor outlet starts decreasing at the beginning of the simulation (though more slowly) instead of after a time delay like in the fixed-bed reactor. The overall time to drop below 25% is, however, much longer than in the fixed bed, at around 250 h compared to 50.

**Figure 11:**
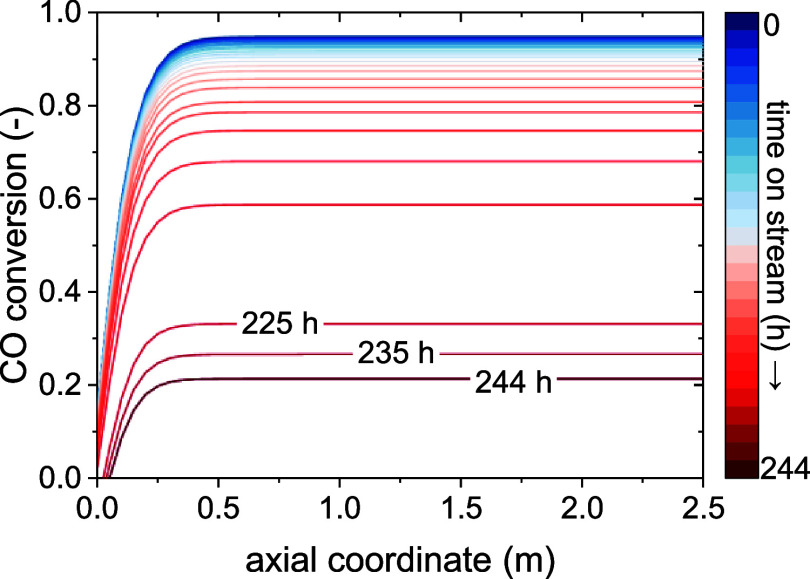
Results of the *Moving Observer model*: conversion loss due to deactivation over the time on stream at a reactor temperature of 300 °C.

The loss of activity of a single particle over the time on stream and the decrease in outlet conversion are reported in Figure 12 as a function of the reactor temperature. The conversion trends in Figure 12 are consistent with experimental literature data.^56^

**Figure 12:**
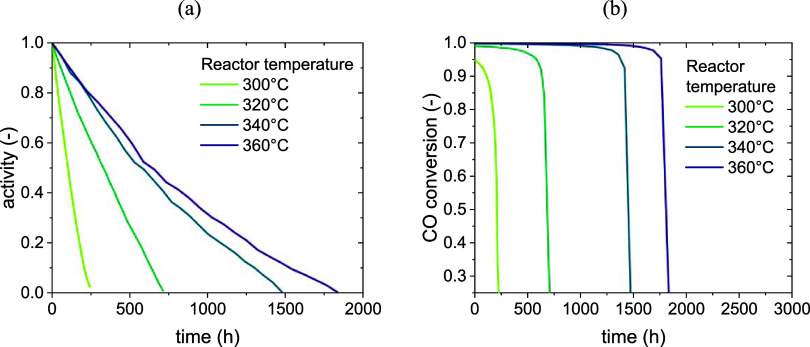
Results of the Moving Observer model: productivity loss due to deactivation (a) and decrease of activity in time (b) (activity defined as per eq 1).

The deactivation of a single particle appears almost linear and much slower than in a fixed bed at the same conditions (250 h against 45 h at 300 °C). The main reasons are listed below.

- *Absence of reaction hotspot.* The temperature in a fixed-bed reactor can rise locally up to almost 600 °C, which increases exponentially the deactivation rate according to the Arrhenius eq 18. Conversely, the perfect mixing of the solid in a fluidized-bed reactor leads to its temperature being uniform in the axial direction, ensuring that the reaction happens at the same temperature regardless of the position in the bed. The lower temperature of the solids ensures a lower deactivation rate.
- *Particle recirculation*. In a fixed bed, a particle localized in the kinetic zone undergoes fast deactivation due to the high content of CO. Once the particles in that zone are completely deactivated, the zone moves forward in the reactor, leading to fast deactivation of a new portion of the bed. In a fluidized bed instead, the particle constantly moves between the reactive zone, in which the high content of CO leads to its deactivation, and the thermodynamic one, in which a large water content prevents this. The relatively small size of the reactive zone compared to the thermodynamic one ensures that the deactivation rate experienced by a particle for most of its time in the bed is close to zero.

Another important effect shown in Figure 12 is the positive effect of an increase in temperature. Different from the fixed-bed case, the decrease of activity of a single particle and of conversion at the reactor outlet proceeds more slowly with increasing temperature. This is an effect of two conflicting trends:

1. On the one hand, an increase in temperature speeds up the deactivation at the same local composition. This means that, roughly, a particle in the reactive zone at 360 °C should deactivate faster than one in the reactive zone at 300 °C, and the same should be valid for a particle in the thermodynamic zone.
2. On the other hand, an increase in temperature leads to a faster methanation kinetics and consumption of CO. This shrinks the reactive zone and causes the particle to spend less time in it, leading to a smaller percentage of time during which the particle experiences a significant deactivation rate. At the same time, the thermodynamic zone increases in size, and its high content of water is responsible for a higher hindering effect on deactivation.

Figure 13 showcases this effect, defining the reactive zone as the reactor length needed to reach 90% conversion. As shown in the picture, this zone is drastically shrunk by an increase in temperature, especially from 300 to 320 °C. The effect is portrayed in the graph at 100, 90, and 75% catalyst activity. It is evident that a lower catalyst activity leads to an increase in the length of the reactive zone due to a slower consumption of CO. This explains the trend observed in Figure 12 that shows a progressively faster decrease in the conversion with time on stream.

**Figure 13:**
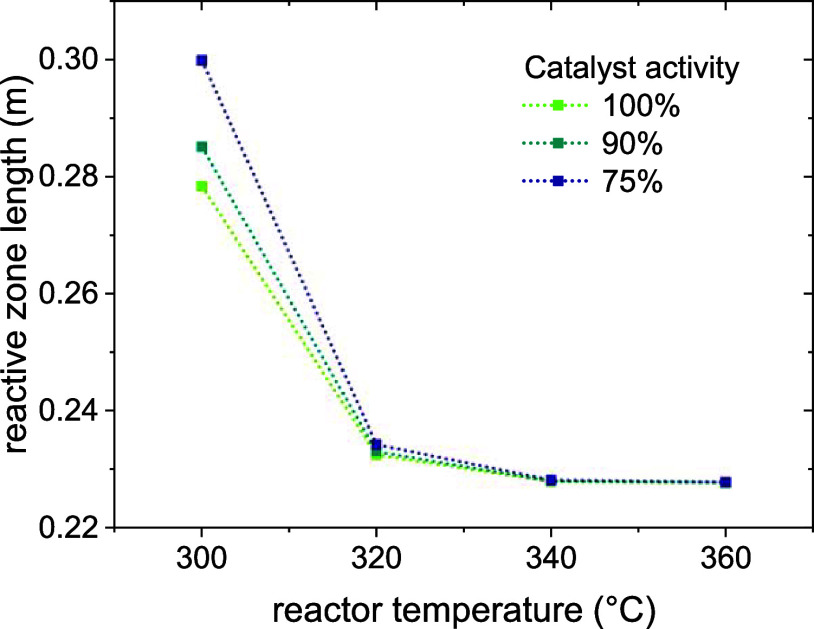
Reactive zone length as a function of the reactor temperature and catalyst activity in a fluidized-bed reactor.

## Conclusions

In this work, an effective and computationally affordable methodology was developed to describe catalyst deactivation by coking in a reactor and the consequent loss of conversion. Reactor dynamics can be modeled through a sequence of steady states, due to the differing time scales between the deactivation and every other phenomenon happening in the reactor (reaction kinetics, transport, etc.). While the global model only requires one differential equation to be solved, it must be coupled with submodels dedicated to deactivation kinetics, calculation of the composition profiles in the reactor, and particle motion (only in the case of fluidized beds).

The *Pseudodynamic* and *Moving Observer* models obtained have been proven effective in assessing the loss of conversion in the reactor due to catalyst deactivation, computationally affordable, and easy to refine, as the accuracy of the results can easily be improved by improving the accuracy of the submodels. The models are also effectively able to deal with a variety of inlet and operating conditions, which makes their use viable in the context of optimization, as the impact of an array of different conditions can be assessed and the optimal conditions can be selected based on the highest reactor performance and minimum impact of deactivation.

As a case study, the behavior of fixed- and fluidized-bed reactors for CO methanation at the same operating conditions of stoichiometric inlet mixture, pressure of 8 bar, and inlet temperature, for the fixed bed, and reactor temperature, for the fluidized bed, was compared. Simulations were run until a 25% outlet conversion of CO was reached, at which point the catalyst was considered completely deactivated. Some interesting trends were observed:

1. The deactivation in fixed-bed reactors behaves as a wave. This is mainly due to the assumption of plug-flow behavior (or anyway limited impact of axial dispersion) for the gas phase, which results in the generation of a hotspot in which the catalyst deactivates much faster than in the rest of the reactor. Once this portion is completely deactivated, no reaction happens there anymore, and the reaction ignition moves further along the axial direction along with the hotspot. This causes the deactivation wave to move forward over the time on stream.
2. The deactivation in fixed-bed reactors speeds up with an increase in temperature. This is an intrinsic kinetic property of the Boudouard reaction, which is mainly responsible for deactivation.
3. Fluidized-bed reactors are much more resistant to deactivation than fixed-bed ones. This is mainly due to the isothermal operation (absence of a hotspot) and to the recirculation of the particles from a small initial zone of the reactor with high CO content, to a larger regeneration zone. It is worth noting that the results for the two different reactor configurations have been obtained for different operating conditions, mainly the value of the space velocity. This may lead to different results in terms of time on stream. However, the main scope of the paper is the definition of a methodology to calculate the time on stream for both reactor types, which can be achieved regardless of the space velocity chosen for the calculations.
4. Deactivation in fluidized-bed reactors appears to be hindered by an increase in temperature, or at least not excessively sped up by it. This is an effect of the faster consumption of CO and consequent smaller active zone of the reactor, which balances the increased deactivation rates by reducing the volume in which deactivation happens.

This work sets the framework for the prediction of the deactivation profiles in fixed- and fluidized-bed reactors over long times on stream. Future works will be focused on improving the model through a refinement of its constituent submodels and the applications to specific case studies with other contaminants apart from CO.

## Supplementary Material



## Notes

Sensitivity analysis on the heat exchange rate inside the reactor (Appendix A), and details of the kinetic model used in the study (Appendix B) (PDF)

## Nomenclature

*A*: reactor cross-section, m^2^
*A*_*B*_: bubble cross-section, m^2^
*Ar*: Archimedes’ number
*a*: catalyst activity (general)
*a*_*j*_: catalyst activity (reaction *j*)
*c*: intensity of correction factor
*ĉ*_*P*_: mass-specific heat of gas mixture, J/Kg·K
*D*_*R*_: reactor diameter m
$D_{\mathrm{ij}}$: mutual diffusion coefficient of species *i* and *j*, m^2^/s
$D_{i}^{m}$: molecular diffusion coefficient of species *i*, m^2^/s
*d*_*B*_: bubble diameter m
*d*_*B*_^0^: initial bubble diameter m
*d*_*B*_^*m*^: maximum bubble diameter m
*d*_*p*_: catalyst particle diameter m
*E*_*d*_: apparent activation energy of deactivation (general), J/mol
*E*_*d,j*_: apparent activation energy of deactivation (reaction *j*), J/mol
*E*_*j*_: activation energy of reaction *j*, J/mol
*F*_*i*_: component *i* molar flow rate, Kmol/s
*F*_*i*_^*B*^: component *i* molar flow rate in bubble phase (fluidized-bed reactor),Kmol/s
*F*_*i*_^*D*^: component *i* molar flow rate in dense phase (fluidized-bed reactor),Kmol/s
*F*_*i*_^D, in^: component *i* molar flow rate at dense phase inlet (fluidized-bed reactor),Kmol/s
*f*_H_2_O_: correction factor for water effect on deactivation
*g*: gravitational acceleration, m/s^2^
*H*: fluidized-bed height m
*H*_mf_: fluidized-bed height at minimum fluidization m
*K*_*j*_^eq^: equilibrium constant of reaction *j* (β depending on mole balance of reaction) Pa^β^
*K*_*k*_: adsorption constant of adsorbate *k*
*K*_*k*_^ref^: adsorption constant of adsorbate *k* at reference temperature -
*k*_*d*_: deactivation kinetic constant (general)
*k*_*d,j*_: deactivation kinetic constant (reaction *j*)
*k*_*d*_^0^: preexponential factor for deactivation kinetic constant
*k*_*j*_: kinetic constant of reaction *j* (β depending on mole balance of reaction), Kmol/s·Kg_c_·Pa^β^
*k*_*j*_^ref^: kinetic constant of reaction *j* at reference temperature (β depending on mole balance of reaction), Kmol/s·Kg_c_·Pa^β^
*L*: reactor length m
*M*_*i*_: molecular weight of species *i*, Kg/mol or Kg/mol
*ṁ*: total mass flow rate, Kg/s
*p*: vector of partial pressures of components Pa or bar
*p*_*i*_: partial pressure of component *i* Pa or bar
*Q*: volumetric flow rate of feed, m^3^/s
*Q*_*B*_: volumetric flow rate of bubble phase (fluidized-bed reactor), m^3^/s
*Q*_*D*_: volumetric flow rate of dense phase (fluidized-bed reactor), m^3^/s
*R*: universal gas constant, J/K·mol
*r*_*d*_: catalyst deactivation rate
*r*_*j*_: reaction rate of reaction *j*, Kmol/m^3^·s
*r*_*j*_^0^: reaction rate of reaction *j* on fresh catalyst, Kmol/m^3^·s
*T*: temperature K
*T*_e_: temperature of external coolant K
*T*_ref_: reference temperature for kinetic constants K
*t*: time on stream h
*U*: global heat transfer coefficient, w/m^2^·K
*u*_*B*_: bubble velocity m
*u*_*G*_^0^: gas superficial velocity,m/s
*V*: reactor volume m^3^
*v*_*i*_^*c*^: critical molar volume of species *i*, m^3^/mol
*W*: width of fixed-bed reactor channels m
*X*: CO/CO_2_ conversion
*X*_0_: CO/CO_2_ conversion on fresh catalyst
*x*: vector of molar fractions of components
*x*_*i*_: molar fraction of species *i*
*y*: generic variable
*z*: axial coordinate in the reactor m
Δ*h*_*k*_^ads^: adsorption heat of adsorbate *k*, J/mol
Δ*h*_*j*_^R^: reaction enthalpy of reaction *j*, J/mol
α_*i*_: apparent reaction order of species *i* in deactivation kinetics -
β: apparent reaction order of activity in deactivation kinetics
Γ_*i*_: interphase mass transfer constant of species *i* (fluidized-bed reactor), 1/s
Γ_*i*_^BC^: bubble-to-cloud mass transfer constant of species *i* (fluidized-bed reactor), 1/s
Γ_*i*_^CD^: cloud-to-emulsion mass transfer constant of species *i* (fluidized-bed reactor), 1/s
δ: bubble fraction (fluidized-bed reactor)
ε: overall void fraction
ε_mf_: overall void fraction at minimum fluidization
μ: gas cinematic viscosity, Pa·s
ν_*ij*_: stoichiometric coefficient of species *i* in reaction *j*
ρ_*pb*_: packed bed density, Kg/m^3^
ρ_*G*_: gas density, Kg/m^3^
