# Supplementary material for: Modeling of the Performance Loss due to Catalyst Deactivation in Fixed- and Fluidized-Bed Reactors
Source: Ind Eng Chem Res. 2025 Aug 26;64(36):17529–42. doi: 10.1021/acs.iecr.5c02248 (PMC12426883; doi:10.1021/acs.iecr.5c02248)
Supplement: Supplementary file 1 [file ie5c02248_si_001.pdf]

SUPPORTING INFORMATION TO:

Modelling of the Performance Loss due to Catalyst Deactivation in Fixed- and Fluidized-bed Reactors

M. Andrea Pappagallo[1,2], Tilman J. Schildhauer[1], Oliver Kröcher[1,2], and Emanuele Moioli[1,3]*

[1] Center for Energy and Environmental Science, Paul Scherrer Institut, Villigen 5232, Switzerland.
[2] Institute of Chemical Sciences and Engineering, École Polytechnique Fédérale de Lausanne, Lausanne 1015, Switzerland.
[3] Dipartimento di Chimica, Materiali e Ingegneria Chimica 'Giulio Natta', Politecnico di Milano, Milano 20133, Italy.

* Corresponding author: emanuele.moioli@polimi.it

Appendix A: sensitivity analysis on the global heat exchange coefficient

Figure S1 shows the effect of applying a multiplicative factor to the global heat exchange coefficient calculated by the heat transfer correlations in the fixed-bed steady-state model.

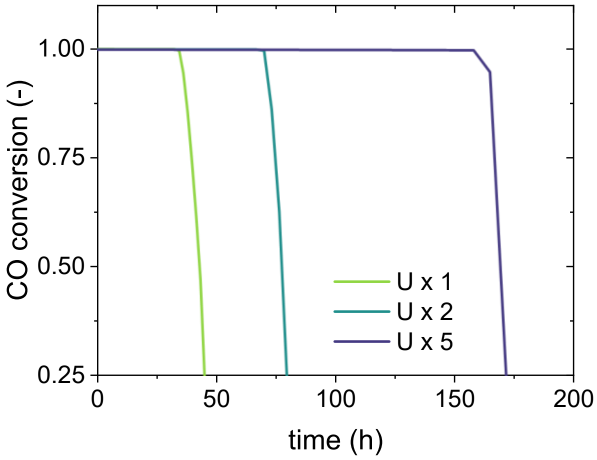

Figure S1: Effect of an increase of the global heat transfer coefficient on the evolution of the outlet conversion over the time on stream (inlet temperature of 300°C).

The increase in the catalyst lifetime is due to the higher heat exchange coefficient lowering the hotspot temperature and reducing the catalyst fraction operating at high temperature. This can be seen in Figure S2, where the evolution of the hotspot for the case at Ux1 and Ux5 is reported. It must be noted that the profiles in Figure S2a coincide with those reported in Figure 9a in the main manuscript.

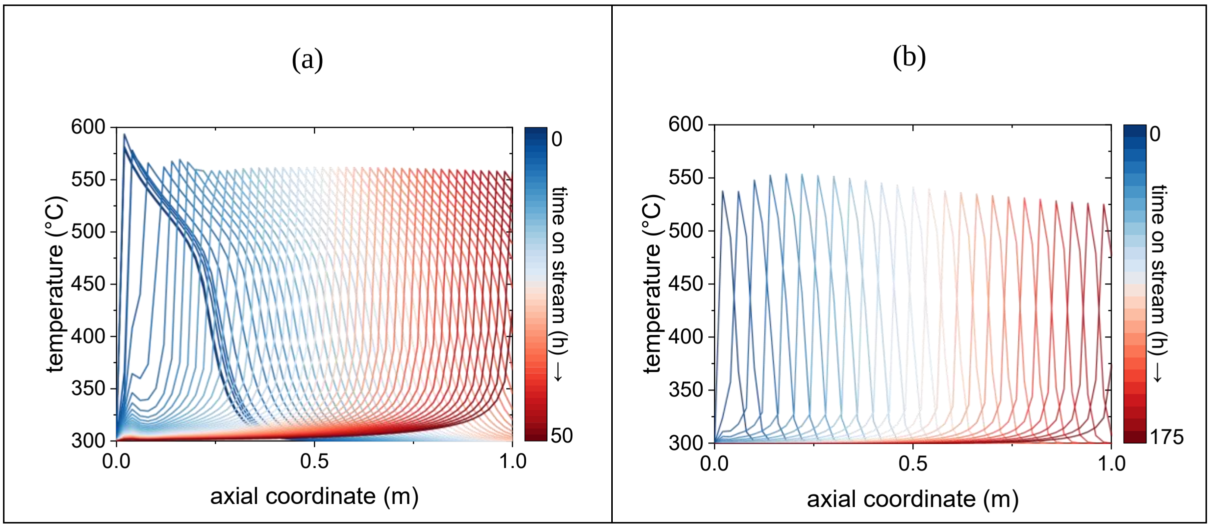

Figure S2: Moving hotspot for the global heat exchange coefficient calculated by the fixed-bed submodel (a) and for a 5-fold increase (b) (inlet temperature of 300°C).

Appendix B: kinetic model parameters

The kinetics used are derived from Koschany et al. [1] (CO_2 methanation reaction) and Kopyscinski et al. [2] (CO methanation and water-gas shift reactions).

Table S1: Parameters of the kinetic model.

Symbol	Parameter	Reaction	Value	Units
k_1^{ref}	preexponential factor	CO methanation	$3.46 \cdot 10^4$	$\frac{\mathrm{kmol}}{\mathrm{s} \cdot \mathrm{kg_c}} \cdot \mathrm{Pa}^{-1}$
k_2^{ref}	preexponential factor	water-gas shift	$7.39 \cdot 10^{-3}$	$\frac{\mathrm{kmol}}{\mathrm{s} \cdot \mathrm{kg_c}} \cdot \mathrm{Pa}^{-2}$
k_3^{ref}	preexponential factor	CO methanation	$1.16 \cdot 10^{-3}$	$\frac{\mathrm{kmol}}{\mathrm{s} \cdot \mathrm{kg_c}} \cdot \mathrm{Pa}^{-1}$
E_1/R	activation energy	CO methanation	$9.32 \cdot 10^3$	$\frac{\mathrm{J}}{\mathrm{mol}}$
E_2/R	activation energy	water-gas shift	32.5	$\frac{\mathrm{J}}{\mathrm{mol}}$
E_3/R	activation energy	CO methanation	14.9	$\frac{\mathrm{J}}{\mathrm{mol}}$
$K_{\mathrm{OH},1}^{\mathrm{ref}}$	preexponential factor (OH adsorption)	CO methanation	0.5	-
$K_{H_2}^{\mathrm{ref}}$	preexponential factor (H adsorption)	CO methanation	0.44	-
$K_{\mathrm{mix}}^{\mathrm{ref}}$	preexponential factor (carbon adsorption)	CO methanation	0.88	-
$\Delta h_\alpha^{\mathrm{ads}}/R$	adsorption enthalpy (OH)	CO methanation	$-2.69 \cdot 10^3$	$\frac{\mathrm{J}}{\mathrm{mol}}$
$\Delta h_C^{\mathrm{ads}}/R$	adsorption enthalpy (H)	CO methanation	$-7.46 \cdot 10^2$	$\frac{\mathrm{J}}{\mathrm{mol}}$
$\Delta h_{\mathrm{OH},2}^{\mathrm{ads}}/R$	adsorption enthalpy (carbon)	CO methanation	$-1.2 \cdot 10^3$	$\frac{\mathrm{J}}{\mathrm{mol}}$
K_α^{ref}	preexponential factor	water-gas shift	0.343	-

K_C^{ref}	preexponential factor (carbon adsorption)	water-gas shift, CO_2 methanation	1.77	-
$K_{\mathrm{OH},2}^{\mathrm{ref}}$	preexponential factor (OH adsorption)	water-gas shift, CO_2 methanation	0.664	-
$\Delta h_\alpha^{\mathrm{ads}}/R$	adsorption enthalpy	water-gas shift	-7.78·10²	$\frac{\mathrm{J}}{\mathrm{mol}}$
$\Delta h_C^{\mathrm{ads}}/R$	adsorption enthalpy (carbon)	water-gas shift, CO_2 methanation	-7.36·10³	$\frac{\mathrm{J}}{\mathrm{mol}}$
$\Delta h_{\mathrm{OH},2}^{\mathrm{ads}}/R$	adsorption enthalpy (OH)	water-gas shift, CO_2 methanation	-8.73·10³	$\frac{\mathrm{J}}{\mathrm{mol}}$

33 Nomenclature

Symbol	Variable	Units
A	reactor cross-section	m^2
A_B	bubble cross-section	m^2
Ar	Archimedes' number	-
a	catalyst activity (general)	-
a_j	catalyst activity (reaction j)	-
c	intensity of correction factor	-
$\hat{c}_P$	mass-specific heat of gas mixture	$\frac{\mathrm{J}}{\mathrm{kg}\cdot\mathrm{K}}$
D_R	reactor diameter	m
$\mathcal{D}_{ij}$	mutual diffusion coefficient of species i and j	$\frac{\mathrm{m}^2}{\mathrm{s}}$
$\mathcal{D}_i^m$	molecular diffusion coefficient of species i	$\frac{\mathrm{m}^2}{\mathrm{s}}$
d_B	bubble diameter	m
d_B^0	initial bubble diameter	m
d_B^m	maximum bubble diameter	m
d_p	catalyst particle diameter	m

E_d	apparent activation energy of deactivation (general)	$\frac{\mathrm{J}}{\mathrm{mol}}$
$E_{d,j}$	apparent activation energy of deactivation (reaction j)	$\frac{\mathrm{J}}{\mathrm{mol}}$
E_j	activation energy of reaction j	$\frac{\mathrm{J}}{\mathrm{mol}}$
F_i	component i molar flowrate	$\frac{\mathrm{kmol}}{\mathrm{s}}$
F_i^B	component i molar flowrate in bubble phase (fluidized-bed reactor)	$\frac{\mathrm{kmol}}{\mathrm{s}}$
F_i^D	component i molar flowrate in dense phase (fluidized-bed reactor)	$\frac{\mathrm{kmol}}{\mathrm{s}}$
$F_i^{\mathrm{D,\,in}}$	component i molar flowrate at dense phase inlet (fluidized-bed reactor)	$\frac{\mathrm{kmol}}{\mathrm{s}}$
f_{H_2O}	correction factor for water effect on deactivation	-
g	gravitational acceleration	$\frac{\mathrm{m}}{\mathrm{s}^2}$
H	fluidized bed height	m
H_{mf}	fluidized bed height at minimum fluidization	m
K_j^{eq}	equilibrium constant of reaction j (β depending on mole balance of reaction)	Pa^{β}
K_k	adsorption constant of adsorbate k	-
K_k^{ref}	adsorption constant of adsorbate k at reference temperature	-
k_d	deactivation kinetic constant (general)	-
$k_{d,j}$	deactivation kinetic constant (reaction j)	-
k_d^0	preexponential factor for deactivation kinetic constant	-
k_j	kinetic constant of reaction j (β depending on mole balance of reaction)	$\frac{\mathrm{kmol}}{\mathrm{s \cdot kg_c}} \cdot \mathrm{Pa}^{\beta}$

k_j^{ref}	kinetic constant of reaction j at reference temperature (β depending on mole balance of reaction)	$\frac{\mathrm{kmol}}{\mathrm{s \cdot kg_c}} \cdot \mathrm{Pa}^{\beta}$
L	reactor length	m
M_i	molecular weight of species i	$\frac{\mathrm{kg}}{\mathrm{mol}}$ or $\frac{\mathrm{kg}}{\mathrm{kmol}}$
$\dot{m}$	total mass flowrate	$\frac{\mathrm{kg}}{\mathrm{s}}$
$\underline{p}$	vector of partial pressures of components	Pa or bar
p_i	partial pressure of component i	Pa or bar
Q	volumetric flowrate of feed	$\frac{\mathrm{m}^3}{\mathrm{s}}$
Q_B	volumetric flowrate of bubble phase (fluidized-bed reactor)	$\frac{\mathrm{m}^3}{\mathrm{s}}$
Q_D	volumetric flowrate of dense phase (fluidized-bed reactor)	$\frac{\mathrm{m}^3}{\mathrm{s}}$
R	universal gas constant	$\frac{\mathrm{J}}{\mathrm{K \cdot mol}}$
r_d	catalyst deactivation rate	-
r_j	reaction rate of reaction j	$\frac{\mathrm{kmol}}{\mathrm{m^3 \cdot s}}$
r_j^0	reaction rate of reaction j on fresh catalyst	$\frac{\mathrm{kmol}}{\mathrm{m^3 \cdot s}}$
T	temperature	K
T_{e}	temperature of external coolant	K
T_{ref}	reference temperature for kinetic constants	K
t	time on stream	h
U	global heat transfer coefficient	$\frac{\mathrm{W}}{\mathrm{m^2 \cdot K}}$

u_B	bubble velocity	m
u_G^0	gas superficial velocity	$\frac{\mathrm{m}}{\mathrm{s}}$
V	reactor volume	m^3
v_i^c	critical molar volume of species i	$\frac{\mathrm{m}^3}{\mathrm{mol}}$
W	width of fixed-bed reactor channels	m
X	CO/CO_2 conversion	-
X_0	CO/CO_2 conversion on fresh catalyst	-
$\underline{x}$	vector of molar fractions of components	-
x_i	molar fraction of species i	-
y	generic variable	-
z	axial coordinate in the reactor	m
$\Delta h_k^{\mathrm{ads}}$	adsorption heat of adsorbate k	$\frac{\mathrm{J}}{\mathrm{mol}}$
Δh_j^{R}	reaction enthalpy of reaction j	$\frac{\mathrm{J}}{\mathrm{mol}}$
α_i	apparent reaction order of species i in deactivation kinetics	-
β	apparent reaction order of activity in deactivation kinetics	
Γ_i	interphase mass transfer constant of species i (fluidized-bed reactor)	$\frac{1}{\mathrm{s}}$
Γ_i^{BC}	bubble-to-cloud mass transfer constant of species i (fluidized-bed reactor)	$\frac{1}{\mathrm{s}}$
Γ_i^{CD}	cloud-to-emulsion mass transfer constant of species i (fluidized-bed reactor)	$\frac{1}{\mathrm{s}}$
δ	bubble fraction (fluidized-bed reactor)	-

ε	overall void fraction	-
$\varepsilon_{\mathrm{mf}}$	overall void fraction at minimum fluidization	-
μ	gas cinematic viscosity	$\mathrm{Pa \cdot s}$
ν_{ij}	stoichiometric coefficient of species i in reaction j	-
ρ_{pb}	packed bed density	$\frac{\mathrm{kg}}{\mathrm{m}^3}$
ρ_G	gas density	$\frac{\mathrm{kg}}{\mathrm{m}^3}$

References

(1) Koschany, F.; Schlereth, D.; Hinrichsen, O. On the Kinetics of the Methanation of Carbon Dioxide on Coprecipitated NiAl(O)x. *Appl Catal B* 2016, *181*, 504–516. https://doi.org/10.1016/j.apcatb.2015.07.026.

(2) Kopyscinski, J.; Schildhauer, T. J.; Vogel, F.; Biollaz, S. M. A.; Wokaun, A. Applying Spatially Resolved Concentration and Temperature Measurements in a Catalytic Plate Reactor for the Kinetic Study of CO Methanation. *J Catal* 2010, *271* (2), 262–279. https://doi.org/10.1016/j.jcat.2010.02.008.
